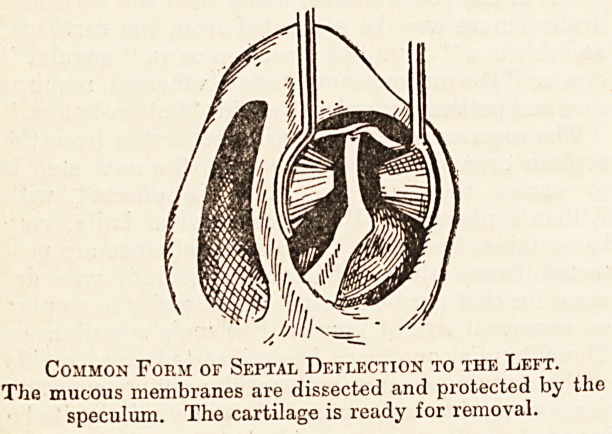# Nasal Obstruction and Operation for Rectifying Obstructive Deformities of the Nasal Septum

**Published:** 1908-03-21

**Authors:** Andrew Wylie

**Affiliations:** Glasgow, Assistant Surgeon, Central London Throat and Ear Hospital.


### Hospital Clinics.

### NASAL OBSTRUCTION AND OPERATION FOR RECTIFYING OBSTRUCTIVE DEFORMITIES OF THE NASAL SEPTUM.

By ANDREW WYLIE, M.D., C.M., Glasgow, Assistant Surgeon, Central London Throat and Ear Hospital.

Nasal obstruction is a very common trouble. Many people suffer from "stuffiness" or "stoppage" of the nose through life without ever thinking of consulting their medical attendant. It is one of the most frequent causes of deafness, and it is this symptom which is often the immediate or direct reason for the patient's seeking advice. Nasal obstruction is responsible for many other troubles, such as parosmia, chronic and acute inflammation of the pharynx and larynx, bronchi and lungs. Further, it not only interferes with the development of those organs, but when severe it is responsible for a state known as "aprosexia" or impaired intellectual development, characterised by lack of ability to concentrate the attention, by dulness of perception, and backwardness in learning, handicapping the sufferer in his struggle through life. It also seriously interferes with general nutrition and growth, and therefore it is of highest importance that the physician should examine the nasal breathway in every form of respiratory impairment.

There are two varieties of nasal obstruction, real and false. The latter is by no means uncommon. It chiefly occurs in young females who, while presenting most of the symptoms, actually possess a perfect breath-way—in fact, too perfect, since the nasal channels may be exceptionally large. Their symptoms may be even exaggerated, with the well-known open lips, flaccid nostrils, vacant expression, and imperfect articulation. Such a state is really due to anæsthesia of the mucous membrane following dryness, or may be due to parental neglect and ignorance. They are unable to feel any current of air in the nose, having a sense of "stuffiness," and firmly believe that they cannot breathe otherwise than through the mouth. Such a state is often difficult to prove to the patient, and still more difficult to remedy, for it is chiefly mental. One must therefore prove to her the freedom of the breathway, and then adopt local treatment to restore a healthy state of the mucous membrane, when nasal breathing will soon be established.

Another very important point is to note whether the obstruction is constant or intermittent, symmetrical or one-sided. Transient and occasional obstruction in adolescents is nearly always functional, and due to variation in the erectile tissue of the inferior turbinal and septum. On the other hand, persistent or constant obstruction, particularly unilateral, indicates some fixed mechanical interference with the breathing. If due to polypi it will vary with posture, and is often associated with occasional epistaxis. Further, it may be only expiratory or inspiratory, according to the position of the polypi. But if due to septal deviation, while possibly varying slightly in degree, the obstruction is always present. The surgeon's next duty will be to localise the course of obstruction, whether it be vestibular, meatal, sinusal or naso-pharyngeal. Vestibular stenosis, apart from deformity and growth, is generally caused by alar collapse, and is easily identified. Temporary hypertrophy of erectile tissue or general hyperæmia readily subsides under cocaine; while varix, permanent hyperplasia of the mucous membrane, growths or bony and cartilaginous deformities are unaffected.

Sinusal trouble is easily recognised by the presence of pus, pain, etc., and by transillumination; while the presence of adenoids is at once ascertained by digital examination, without which no diagnosis is complete. Having decided that the obstruction is not due to any cause other than septal deformity, one's next duty is to define the exact site and nature of the deformity, and then to take the steps necessary for its rectification. Most spontaneous deviations from the normal involve the bony portion of the septum, while displacements and errors in the cartilage are more likely to be traumatic in origin.

The bony septum consists of two distinct portions, a lower or vomerine element, which is developed by perichondrial ossification with considerable thickness of diploic tissue intervening, and an upper or ethmoid element, which is solid, and is developed by endochondrial ossification. At the junction of these elements is a ridge or suture, which slopes upwards and backwards, and is often the seat of spurs uni- or bi-lateral, bony or cartilaginous. By reason of the spur being situated in a vomerine part of the septum, and this being bilaminar, its removal does not necessarily cause a perforation. The deformity which most frequently calls for correction is that known as "crumpling of the septum," which may be horizontal, vertical, or even mixed. This involves the whole thickness of the septum, and presents a convexity and a corresponding concavity.

### Ætiology.

In a thousand cases complaining of "stuffiness" which I examined at the Central London Throat and Ear Hospital I found 80 per cent. with deviation of the septum to the left, and all confessed to holding their handkerchiefs in their right hand when blowing their nose. Eight per cent. had deviations to the right, and were mostly left-handed people. The remaining 12 per cent. of deviations were in ambidextrous people. Some rhinologists attribute the deviations to habitual sleeping on the opposite side to the deviation with the nose buried in the pillow, but this certainly does not explain the horizontal crumpling, although it may possibly be responsible for cartilaginous deviation. Other causes are blows, especially in school during the adolescent period, and they chiefly involve the cartilage; falls, when an infant, on the floor or against furniture; foreign bodies, as shells, peas, etc., put up one nostril by children when the nasal framework is developing; turbinal hypertrophy, growths, polypi, etc., tend to make room for themselves by pushing the septum towards the free side.

Whether traumatism is directly or indirectly a causal factor there is no doubt whatever that some influence is at work during the development and growth of this organ. Further, that it is essentially a disease of civilisation, since septal errors are extremely rare in aboriginals, and those who use no handkerchief, beds, and pillows. The growth and development of the skull is such a complicated process that any interference with one part is bound to influence the other parts more or less—that is, the development of the nostrils, the maxillary antra, the palate and the teeth are all dependent upon one another; if disturbance takes place in one the whole fabric is adversely affected. Hence deficient nutrition and ossification must obviously be responsible for distortion of this as well as other parts of the body framework. Premature ossification of a suture must distort adjacent structures where growth is not completed. Both premature ossification of the interpalatal and maxillary sutures and the gothic-arched palate are said to cause septal crumpling, but these may with equal reason be interpreted as the consequences, since there may be delayed as well as premature ossification. It is remarkable how few people have a straight septum. Take one hundred people from the street, and I believe that about ninety will have some deviation or thickening of the septum. But it may be very slight, and cause no trouble. As already stated, a large proportion of deviations of the septum are towards the left side—*i.e.* the convex side in the left nostril and concave in the right. The left nostril is therefore "stuffy," and the right nostril roomy and free, unless its inferior turbinal undergoes compensatory hypertrophy, and in these cases one often finds that the left ear is slightly deaf when the obstruction is severe.

Deformities of the septum are of all kinds:

1. Simple deviations towards one side, chiefly the left, and cartilaginous in character.
2. Those which obstruct both nostrils more or less by being deviated in two directions or being very thick in one part and thin at another, called "corkscrew deviations."
3. Deviations which are thickened in one place and thin at another, and project into both nostrils (bilaminar) symmetrically or in an irregular manner like a piece of crushed paper, called "crumpled septum."
4. Deviations which have a spur projecting from them; which causes great difficulty in the operation.
5. Deviations which are caused by a severe blow, when a fracture at the osseo-chondral suture has taken place and the cartilaginous septum projects and causes complete stoppage of one nostril. This is sometimes loose, and constitutes dislocation of the cartilage.

Simple deviations to one side or the other are easily rectified, and uncomplicated spurs can be easily shaved off; but these simple cases are, unfortunately, not the common forms. Those which come for treatment are usually very severe, the obstruction is often in both nostrils, due to a double curve of the septum like a letter S, and is often attended with facial disfigurement which prompts the search for advice. To overcome these defects and restore free nasal respiration has been a problem to rhinologists for many years.

The method employed until lately was to remove a part of the whole thickness of the septum with the obstruction, and if this was done thoroughly, free nasal respiration was restored; but, of course, a perforation was generally made. Perforations of the septum are spontaneously caused by syphilis, lupus, tuberculosis, "nose picking," and abscess following inflammation. In many cases, especially when small, and situated posteriorly, the patients do not know they have a perforation, since there are no troublesome symptoms and no whistling sound is heard or felt. On the other hand, perforations often cause crusts to form which are attended by peculiar disagreeable subjective odours and foul breath, which cause the patient to resort to habitual douching the nose with antiseptic lotions, eventually leading to middle-ear trouble. For these reasons the simple operation of wholesale and indiscriminate removal is not recommended. Although perforations in a fully developed septum may be less serious than in a growing one, and may not give rise to any symptoms, still it is better avoided, therefore any operation which does not risk their formation is to be preferred. As already mentioned, the lower or vomerine septum is bilaminar, therefore a direct slicing operation as with a spokeshave or saw may only involve one side, and consequently does not perforate; but the case may not be so accommodating. When the deviation is osseo-cartilaginous and vertical Moure's operation (which consists of cutting through the septum from both sides with sharp cutting or "button-holing" pliers and subsequent straightening) may be followed by excellent results and no perforation. But this applies only to a very small percentage of cases, and must be strictly limited to that variety which is suitable. It is certainly a conservative measure, although it is liable to failure by reason of sepsis or relapse. Moure's operation should be recommended in childhood or early life when the aperture is too small for a thorough submucous resection.

Before going into surgical details I wish to emphasise a very important point, namely, the evil of "over-operating" on the nasal septum. Stuffiness of the nose is, as I said, a very common trouble, especially in young men and girls entering the adolescent stage of life, and these should hardly ever be operated on; in fact, they should be treated as little as possible; active local interference is disastrous. Continual douching and spraying may create an anæsthesia of the mucous membrane of the nostril which may interfere with natural nasal respiration throughout life. Operations in children are also to be avoided unless the disease allows no breathway. The nasal cavities are not fully developed until puberty is well passed.

### Treatment.

The operation which has within the last few years become so general was brought before the medical profession by Killian, of Freiburg, and is called, for want of a better name, "Submucous Resection." This operation is said to have been performed as early as 1860, but Killian's name has been closely associated with it, and later perfection has followed at the hands of British and American rhinologists. The idea of the submucous resection is to excise all the skeletal or cartilaginous and bony parts of the septum which cause obstruction, while at the same time preserving intact the mucous membrane and submucous tissue on each side. The advantages of this operation are that the obstruction is at once removed and free respiration assured; that the obstruction cannot return; that no perforation (however small) is made; and, finally, that as the normal mucous membrane remains intact no crusts or scabs result.

The operation appears very easy on paper, and in simple cases it is very easy to perform, but since no two cases are alike, the surgeon must always be ready for one which is troublesome, when all his ingenuity, skill, patience, and judgment are put to the test.

### Anæsthesia.

This depends on the position in which the surgeon wishes the patient to be placed. The easiest method is to have the patient sitting in a chair opposite him, and is the one most commonly advocated, since it possesses the advantages (1) of being that in which he is accustomed to examine the patient; it also (2) brings the obstructing parts well into view; also (3) the patient can help the surgeon by moving the head as directed; (4) it is an easier position for the surgeon to work; (5) it is adapted to local, which is safer than general, anæsthesia. The disadvantages are: (1) The discomfort the patient is subjected to when undergoing a prolonged operation, even although there is no real pain; (2) the speed at which the surgeon must operate to be finished before the local anæsthesia loses its effect; (3) the probable collapse of the patient, caused by the mental impression and the depressing effect of the local anæsthesia.

Every surgeon has his own particular method. Those who prefer the upright position must anæsthetise very thoroughly by injecting 10 ♏ of a ½-per-cent. solution of cocaine in normal saline with two drops of 1-1000 solution of adrenalin into each side of the septum with a hypodermic syringe. My usual method is to perform the operation in the recumbent position under a general anæsthetic, with the head and shoulders well raised, so as to operate under a reflected light.

The advantages are: (1) That the operator does not need to hurry, and can work with comfort and deliberation; (2) the patient is unconscious, and has no mental strain nor shock. But the disadvantages are that: (1) There is always an element of danger in general anæsthesia, especially as in nasal cases blood is liable to trickle down the pharynx into the larynx; (2) the surgeon does not see the floor of the nose so well, and may overlook some projecting obstructive mass; (3) the position of the surgeon in bending over the patient is not one of mechanical advantage.

I generally, for hæmostatic purposes, half an hour previous to administering the chloroform, plug each nares thoroughly with cotton wool soaked in a 5-per cent. solution of cocaine with equal parts of adrenalin solution 1-1000. This absolutely controls hæmorrhage. A further spraying with weak phenol and Dobell's solution ensures temporary asepsis. While the deeper parts of the nostrils and the naso-pharynx may be normally sterile, it must be remembered that the mouth is never so, and being so close it is necessary to thoroughly cleanse it, together with the lips, moustache and adjacent parts to diminish the risk of sepsis. The teeth should also previously be attended to by a dentist.

### Instruments.

These are multiplying with great rapidity. Every operator thinks his own devices for removing certain obstructions are the best, but a skilful operator needs but a small armamentarium, consisting of: 1, Small nasal speculum; 2, a large one, such as Killian's; 3, sharp knife; 4, blunt-pointed knife; 5, elevator; 6, Killian's plough or Ballenger's swivel knife; 7, Jansen-Middleton or Heath forceps; 8, bent chisel; 9, a strong pair of catch forceps.

### Operation.

The first incision is made with a small knife into the mucous membrane of the convex side from the roof to the floor of the vestibule, and the incision must be carried through the perichondrium to the cartilage itself. With the blunt elevator the mucous membrane is separated from the cartilage along the whole of the convex side. This is easily done in a simple deviation, especially if the surgeon makes sure that the perichondrium is separated also, as it is stripped with ease, and is useful subsequently when the mucous membranes form the bony septum. It is not so easy when a spur or ridge is growing.

The next step is to cut the cartilage with a small blunt pointed tenotomy knife in the same line as the original incision, taking care not to pierce the mucous membrane on the other side (concave side), which must be kept intact. The fore or little finger in the concave nostril is a good guide. The mucous membrane of the concave side, along with the perichondrium, must now be separated from the cartilage, and this in a "crumpled" septum or an "angular" one, or if the mucous membrane is adherent, requires care and patience in order to avoid "button-holing."

The mucous membrane being separated from the septum over all ridges and angles, the next step is to excise the cartilage. This is effected with Killian's plough or Ballenger's swivel knife, care being taken that these mucous membranes are protected from injury. Killian's speculum was devised for that purpose, but I find it easier to employ an abnormal size of Lennox Browne's speculum or Thudichum's, one blade being inserted on each side of the septum, or, even better still, a copper guard on the convex side and a guard of my own design to be inserted on the concave side between the mucous membrane and the septum, an assistant holding it firmly. A large piece of the cartilage must now be removed; one can scarcely remove too much; if only a small piece is removed, or a "window" made in it, at some later date the resilient curved edges of the cartilage will spring out and spoil our work.

Ballenger's knife is excellent, if pushed well upwards and backwards, then downwards until the lower margin of the vomer is reached and pulled out along its maxillary border—a large piece of cartilage is thus removed, and the obstruction in a simple case is overcome; but as a rule there are still overhanging

Mucous Membranes Dissected from Cartilage.
The convex one on the right held by retractor and the concave by author's guard.

pieces, which should be removed with Jansen's or Heath's forceps, the two mucous membranes being guarded as before. The lower border or maxillary crest must now be thoroughly inspected, for if this, frequently prominent part, is left our operation is spoilt. It is easily seen when the patient is sitting up, but is more difficult when in the recumbent position. To remove the "crest" in the maxillæ we must still protect the mucous membranes from injury by the speculum or guards, and gently chip it away with the bent chisel. To finish the operation see that the septum behind is free—that no obstructive part intervenes; then withdraw the guards and allow the mucous membranes to fall together in the middle line. The one on the concave side should be intact and the one on the convex side be cut only at its anterior and lower border. This can be easily stitched by means of catgut sutures and a curved needle of an appropriate shape or size.

Common Form of Septal Deflection to the Left.
The mucous membranes are dissected and protected by the speculum. The cartilage is ready for removal.

Most rhinologists pack the nares to keep the mucous membranes in their place and in apposition, but my experience is that when the packing is removed it has a tendency to draw the mucous membrane with it; I, therefore, rarely pack, so that the blood and serum which oozes out has a free drainage and the patients are not subjected to the discomfort of a plugged nostril even for twenty-four hours.

For fully a week or two inflammatory reaction with swelling ensues and no relief is felt; but when these symptoms disappear, the result compensates for all the discomfort: the freedom in breathing, the marked change in the voice, and the way the head is held denote a new man bodily and mentally.

The time occupied varies with the extent and shape of the obstruction, the ease with which the soft tissues are detached, the amount of hæmorrhage, the intelligence and self-control of the patient, and the dexterity and patience of the surgeon.

The most suitable age for operation is between 17 and 30. It is essential to have a period of life of structural activity. I would not advise the operation in old people who have become accustomed to the obstruction and afford no evidence of inconvenience, since soft structures have then a marked tendency to atrophy, with a gradual enlargement of a previously narrow breathway.

The operation has not been practised for a sufficiently long period to enable any definite conclusions to be drawn as to its expediency in very early life—at least before puberty. Judging, however, by the effect of very early perforation of the bony septum, by syphilis, for instance, disfigurement may follow; but later perforations, traumatic and surgical, when confined to the cartilage, are not followed by deformity, and, in fact, tend to close up by periosteal deposit.

This last change can always be looked for in the vomer, but it is somewhat doubtful in the ethmoid element. The question seems to bear upon the condition of the ethmo-vomerine suture, which is cartilagious until 18-20. Along this line bone-growth continues, and if not extensively interfered with will give rise to no permanent disfigurement.

With very young children it is a disputed point, for the operation might influence a full and symmetrical development of the nose. I have no hesitation in advising the operation if the deviation is very pronounced and greatly obstructs respiration so as to interfere with the development of the chest, and to impair the general health. Moure's operation may be preferable in childhood.

### After Results.

It has been proved that no deformity of the nose results if a sufficiency of cartilage be left. Many gross and obvious deformities of the nose are also improved, if not entirely cured, by the operation. Our boast in this operation is that the mucous membrane, perichondrium, and periosteum are left intact, that there is no loss of ciliated and squamous epithelium; consequently no crusts or scabs form, and no long after-healing process. This operation is not recommended when there is syphilis, tubercle, lupus, or malignant disease of the nose. It in many cases can be performed easily and speedily; but perfection is more to be aimed at than speed, and with the patient under a general anæsthetic it is best for the surgeon to be patient and careful, for one hasty touch may cause a slight tear, and no amount of regrets or excuses will make that septum intact again.

The ultimate successful result of the operation must be free respiration on both sides, with an intact, but soft, firm septum.

## Figures and Tables

**Figure f1:**
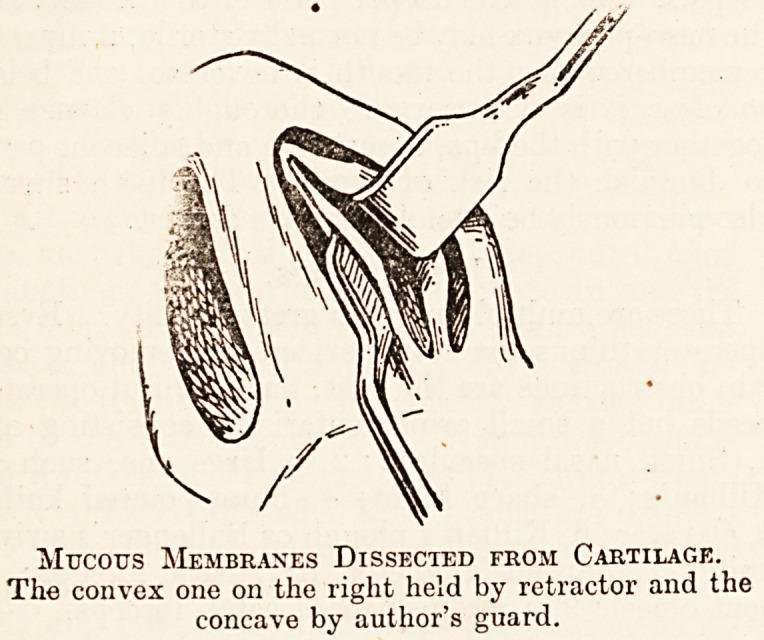


**Figure f2:**